# Steroidal alkaloids and conessine from the medicinal plant *Holarrhena antidysenterica* restore antibiotic efficacy in a *Galleria mellonella* model of multidrug-resistant *Pseudomonas aeruginosa* infection

**DOI:** 10.1186/s12906-018-2348-9

**Published:** 2018-10-19

**Authors:** Thanyaluck Siriyong, Supayang Piyawan Voravuthikunchai, Peter John Coote

**Affiliations:** 1 Department of Microbiology, Faculty of Science and Natural Product Research Center of Excellence, Prince of Songkla University, Songkhla, Thailand; 2 Biomedical Sciences Research Complex, School of Biology, University of St Andrews, The North Haugh, St Andrews, Fife, UK

**Keywords:** Conessine, Efflux pump inhibitor, *Galleria mellonella*, *Holarrhena antidysenterica*, Mex efflux systems

## Abstract

**Background:**

This study aimed to evaluate the efficacy of combinations of steroidal alkaloids and conessine from the Thai medicinal plant *Holarrhena antidysenterica* with antibiotics against *Pseudomonas aeruginosa* strains possessing different efflux-pump-mediated multidrug-resistant (MDR) phenotypes in a *Galleria mellonella* infection model.

**Methods:**

*P. aeruginosa* strains with defined mutations that result in the overexpression of the MexAB-OprM, MexCD-OprJ and MexEF-OprN efflux pumps, and a strain with all three of these pumps deleted, were used. In vitro, the effect of combinations of steroidal alkaloids and conessine with antibiotics was compared with antibiotic treatment alone via MIC determination and time-kill assays. Efficacy of combinations of the steroidal alkaloids and conessine with levofloxacin were compared with monotherapies against infections in *G. mellonella* larvae by measuring larval mortality and bacterial burden.

**Results:**

Combination therapies of conessine or steroidal alkaloids with levofloxacin enhanced bacterial inhibition in vitro and restored antibiotic efficacy in vivo compared to the constituent monotherapies. Neither conessine nor the steroidal alkaloids induced any detectable toxicity in *G. mellonella* larvae. The enhanced efficacy of the combination treatments was most pronounced with conessine and correlated with reduced larval burden of infecting *P. aeruginosa*. Notably, the enhanced efficacy of conessine/levofloxacin combinations was only detected in the parent strain and strains that overexpressed the MexAB-OprM or MexEF-OprN efflux systems.

**Conclusions:**

Steroidal alkaloids from *Holarrhena antidysenterica*, and particularly the principal active ingredient conessine, restored levofloxacin efficacy against resistant *P. aeruginosa* strains possessing efflux-mediated MDR phenotypes. The compounds should be investigated further as a potential novel therapy.

**Electronic supplementary material:**

The online version of this article (10.1186/s12906-018-2348-9) contains supplementary material, which is available to authorized users.

## Background

Global emergence of multidrug-resistant (MDR) *Pseudomonas aeruginosa* is now a growing threat to antibiotic therapy. Chromosomally encoded antibiotic efflux mechanisms greatly contribute to antibiotic resistance in this organism, in particular, the multidrug efflux pumps of the resistance-nodulation-division (RND) superfamily such as MexAB-OprM, MexCD-OprJ, MexEF-OprN, and MexXY-OprM [1, 2]. Clinical isolates of *P. aeruginosa* are often identified with mutations in regulatory genes that result in the over-expression of these RND efflux pumps and confer a MDR phenotype [1, 3]. The MDR phenotype occurs due to the broad and overlapping range of antibiotic substrates that the RND pumps efflux, particularly MexAB-OprM [3]. Thus, multidrug efflux pumps represent potential targets for the development of novel treatment regimens for MDR *P. aeruginosa*.

The use of efflux pump inhibitors (EPIs) as adjuncts in combination therapies with existing antibiotics is a potential strategy for preventing efflux-mediated resistance and restoring antibiotic efficacy [4]. Many synthetic compounds such as phenylalanine-arginine β-naphthylamide (PAβN), carbonyl cyanide *m*-chlorophenylhydrazone (CCCP), quinoline derivatives, and 1-(1-naphthylmethyl)-piperazine (NMP) display efflux pump inhibitory activity however none have yet been developed for clinical application [5, 6]. Some existing drugs have also been found to possess efflux-pump inhibitory properties and could be repurposed as resistance modifying agents when administered in combination with antibiotics. Examples include trimethoprim and the selective serotonin reuptake inhibitor sertraline [5, 7, 8].

Currently, a number of naturally-occurring compounds with possible EPI activity including berberine [9], curcumin [10, 11], and p-coumaric acid [12] are being actively researched for potential future application [13, 14]. Extracts made from the stem bark of the plant *Holarrhena antidysenterica* (Linn) Wallich belonging to the family Apocynaceae, are used in Thai and Ayurvedic traditional medicine to treat amoebic dysentery and diarrhoea [15, 16]. Steroidal alkaloids present within the extracts possess antibacterial, anti-diarrhoeal and astringent properties [15, 16]. Previous studies indicated that *H. antidysenterica* extract, and the principal active ingredient alone (the steroidal alkaloid conessine [16]), were able to restore the inhibitory activity of novobiocin and rifampicin against extensively drug-resistant *Acinetobacter baumannii* in vitro [17–19]. Exposure to either *H. antidysenterica* extract, or conessine alone, at concentrations that did not result in increased membrane disruption, resulted in accumulation of the fluorescent dye Pyronin Y suggesting that both treatments may have EPI-like properties [19]. Moreover, exposure to conessine restored the MICs of antibiotics against a *P. aeruginosa* strain over-expressing MexAB-OprM to values comparable with the wild-type parent and conessine induced the accumulation of Hoechst 33342 in cells over-expressing the same efflux pump [20].

Previous work has employed *Galleria mellonella* larvae to demonstrate enhanced efficacy of putative EPIs in combination with antibiotics versus infections by MDR strains of *P. aeruginosa* that over-express various RND efflux pumps [10, 21]. The aim of this study was to evaluate the efficacy of combinations of antibiotics with either *H. antidysenterica* extracts, or the steroidal alkaloid conessine, using a *G. mellonella* infection model to determine if these combinations were toxic, or could treat successfully, infections with *P. aeruginosa* strains possessing an efflux-pump dependent MDR phenotype due to over-expression of either MexAB-OprM, MexCD-OprJ or MexEF-OprN.

## Methods

### Bacteria and growth media

*P. aeruginosa* strains PAM1020 (PAO1 prototroph, wild-type); PAM1032 (*nalB*), MexAB-OprM overexpressed; PAM1033 (*nfxB*), MexCD-OprJ overexpressed; PAM1034 (*nfxC*), MexEF-OprN overexpressed; and PAM1626 (*∆mex*), MexAB-OprM, MexCD-OprJ and MexEF-OprN deletion, were a generous gift from Dr. Olga Lomovskaya, Rempex Pharmaceuticals, USA [22]. The strains were grown to mid-log phase in Mueller–Hinton broth (MHB; Merck, Darmstadt, Germany) at 37 °C with shaking (at 200 rpm) to prepare inocula for antibiotic susceptibility testing in vitro and efficacy testing in vivo.

### Chemicals and *G. mellonella* larvae

Conessine and the antibiotics ceftazidime, piperacillin, meropenem, amikacin, and levofloxacin were purchased from Sigma–Aldrich Ltd. (Dorset, UK). Stock solutions of antibiotics were prepared in sterile deionized water. Conessine was made in 70% Tween 80 (Sigma–Aldrich Ltd., Dorset, UK) and 30% ethanol. Sub-stocks of all antibiotics and conessine used in experiments were dissolved in sterile deionized water. *G. mellonella* larvae were obtained from UK Waxworms Ltd. (Sheffield, UK). Stock solutions of steroidal alkaloids from *H. antidysenterica* bark extract were prepared in 100% ethanol as previously described [15]. Briefly, fresh barks were washed with distilled water and dried at 60 °C overnight. Finely powdered bark (4 kg) was macerated with 95% ethanol (1:2 *w*/*v*) to obtain an alcohol extract, evaporated to dryness, and then suspended in methanol. The suspension was adjusted to pH = 3.2 by addition of HCl (2 M). The solution was basified to pH = 9 with NaOH (2 M) and then extracted with chloroform (0.4 L × 5) to obtain the total alkaloids (25.63 g). Sub-stocks used in experiments were made in sterile deionized water.

### Antibiotic susceptibility testing

MICs of antibiotics (ceftazidime, piperacillin, meropenem, amikacin, and levofloxacin), conessine, or steroidal alkaloids against each of the *P. aeruginosa* strains were determined in 96-well microplates as previously described [23]. Briefly, doubling dilutions of each antibiotic, conessine or steroidal alkaloids were prepared in MHB and subsequently inoculated with 1.0 × 10^6^ cfu/mL of *P. aeruginosa*. The effect of conessine or steroidal alkaloids in combination with each antibiotic was measured by first preparing 96-well plates with doubling dilutions of each antibiotic in MHB as described above. Following this, single concentrations of either steroidal extracts (1024 mg/L) or conessine (MIC_0.5_–32 mg/L or MIC_0.25_–16 mg/L) were added to each well prior to inoculation with *P. aeruginosa* as before. Microplates were incubated at 37 °C and the MIC was defined as the concentration(s) present in the first optically clear well after 24 h. Each experiment was performed at least twice. Fractional inhibitory concentration index (FICI) = (MIC_drug + steroidal alkaloids or conessine_/MIC_steroidal alkaloids or conessine_) + (MIC_drug + steroidal alkaloids or conessine_/MIC_antibiotic_) were calculated for each combination and synergy was defined as FICI ≤0.5 [24].

### Determination of *P. aeruginosa* viability

*P. aeruginosa* viability was assessed after 24 h exposure to levofloxacin (0.125, 1, 2 mg/L), conessine (32 mg/L), or steroidal alkaloids (1024 mg/L), and combinations of levofloxacin with conessine or steroidal alkaloids. Aliquots of tested compounds were added to 96-well microplate wells containing MHB, while identical volumes of sterile water were added to control wells. Microplates were inoculated with 1.0 × 10^6^ cfu/mL of *P. aeruginosa* and the plates were incubated at 37 °C for 24 h. Subsequently, viable bacteria were determined by serial dilution in MHB and plating on nutrient agar (NA; Merck, Darmstadt, Germany). Plates were incubated at 37 °C for 24 h prior to counting colonies. Each treatment was replicated in quadruplicate and a mean value was calculated. Synergy was defined as a ≥ 2-log_10_ decrease in colony count at 24 h by the combination therapies compared with the most effective single treatments, as well as a ≥ 2-log_10_ decrease in colony count compared with the starting inoculum [25].

### *G. mellonella* model of *P. aeruginosa* infection

*G. mellonella* at their final instar larval stage were kept at room temperature in darkness. Larvae weighing within the range of 250 to 350 mg were selected for each experiment to ensure consistency in subsequent drug administrations and were used within 1 week of receipt.

Efficacy of conessine or steroidal alkaloids in combination with levofloxacin versus *G. mellonella* larvae infected with the *P. aeruginosa* strains was carried out exactly as described previously [21, 23, 26]. Briefly, groups of 15 larvae were infected with an inoculum of 2.5 × 10^3^ cfu/mL of *P. aeruginosa* cells. Treatments with three doses of conessine or steroidal alkaloids, levofloxacin, and combinations of these compounds were administered 2, 4, and 6 h post-infection. Levofloxacin doses of 1 and 0.05 mg/kg were used for *P. aeruginosa* PAM1020 and PAM1626 respectively and a dose of 5 mg/kg of levofloxacin was utilized for PAM1032, PAM1033 and PAM1034 [21]. Conessine was administered at 50 mg/kg and steroidal alkaloids at 50, 100 and 200 mg/kg in all tested strains. The experiments were repeated in triplicate using larvae from different batches and the data from these replicate experiments were pooled to give *n* = 45. Survival data were plotted using the Kaplan–Meier method [27] and comparisons made between groups using the log-rank test [28]. In all comparisons with the negative control it was the uninfected control (rather than the unmanipulated control) that was used. Holm’s correction was applied to account for multiple comparisons in all tests and *P* ≤ 0.05 was considered significant [29].

### *G. mellonella* haemolymph burden

Larval burden of five randomly selected caterpillars from each treatment group was measured at 24 h intervals exactly as described previously [10, 21, 26]. Groups of 30 larvae were infected with 2.5 × 10^3^ cfu/mL of *P. aeruginosa*. Conessine (50 mg/kg) or steroidal alkaloids (50, 100 or 200 mg/kg), levofloxacin (1 or 5 mg/kg), and the combinations were administered at 2, 4, and 6 h post-infection. Larvae were incubated in Petri dishes at 37 °C. The detection limit for this assay was 100 cfu/mL of larval homogenate.

## Results

### *H. antidysenterica* steroidal alkaloids and conessine increase the susceptibility of *P. aeruginosa* to antibiotics in vitro

Alone, the steroidal alkaloids had no inhibitory action and the MIC for the parent strain and the strains overexpressing each of the efflux systems was > 1024 mg/L. However, deletion of all three efflux systems (PAM1626) did induce some sensitivity as the MIC for this strain was 256 mg/L. In contrast, the MIC for conessine was identical for all of the strains tested: 64 mg/L, suggesting that conessine is not a substrate for the MexAB-OprM, MexCD-OprJ, and MexEF-OprN efflux pumps.

The susceptibility of the *P. aeruginosa* strains to a group of anti-pseudomonal antibiotics alone or in the presence of steroidal alkaloids (128 or 1024 mg/L) or conessine (32 mg/L) is shown in Table 1. In this study, steroidal alkaloids extract at 1024 mg/L contained approximately 37 mg/L conessine content [19]. The strain overexpressing the MexAB-OprM efflux pump (PAM1032) was more resistant to ceftazidime, piperacillin, meropenem and levofloxacin in comparison to the parent strain (PAM1020). In contrast, the strains overexpressing the MexCD-OprJ (PAM1033) and MexEF-OprN (PAM1034) efflux systems displayed resistance to levofloxacin only. Deletion of all three efflux systems (PAM1626) resulted in increased susceptibility to all of the antibiotics compared to the parent strain. These results are in accordance with published reports on the substrate specificity of these efflux pumps [1, 21].

**Table 1:** MICs of five antibiotics alone and in the presence of *Holarrhena antidysenterica* steroidal alkaloids or conessine against *P. aeruginosa* PAM1020, 1032, 1033, 1034 and 1626

Strain	Drug	Drug MIC (mg/L) with			FIC index^b^	
		Alone	Steroidal alkaloids^a^	Conessine^a^	Drug + Steroidal alkaloids	Drug + Conessine
PAM1020	CAZ	1	0.5	1	1.50	1.50
	PIP	2	1	2	1.50	1.50
	MEM	0.5	0.5	0.25	2.00	1.00
	LVX	0.5	0.5	0.25	2.00	1.00
	AMK	1	0.5	0.5	1.50	1.00
PAM1032	CAZ	4	2	2	1.50	1.00
	PIP	16	16	8	2.00	1.00
	MEM	4	4	2	2.00	1.00
	LVX	2	1	1	1.50	1.00
	AMK	1	0.5	0.5	1.50	1.00
PAM1033	CAZ	1	0.5	1	1.50	1.50
	PIP	2	1	2	1.50	1.50
	MEM	0.5	0.25	0.5	1.50	1.50
	LVX	4	4	4	2.00	1.50
	AMK	0.5	0.25	0.5	1.50	1.50
PAM1034	CAZ	1	1	0.5	2.00	1.00
	PIP	1	0.5	0.03125	1.50	0.53
	MEM	0.5	0.5	0.125	2.00	0.75
	LVX	4	2	0.25	1.50	0.56
	AMK	0.5	0.25	0.5	1.50	1.50
PAM1626	CAZ	0.5	0.5	0.25	1.50	1.00
	PIP	0.5	0.25	0.5	1.00	1.50
	MEM	0.0625	0.0625	0.0625	1.50	1.50
	LVX	0.03125	0.00781	0.00391	0.75	0.63
	AMK	0.5	0.25	0.25	1.00	1.00

*CAZ* ceftazidime, *PIP* piperacillin, *MEM* meropenem, *LVX* levofloxacin, *AMK* amikacin

^a^The concentration of steroidal alkaloids or conessine added to each well reflected the previously characterized MICs and were selected to be lower than the MIC for each strain: PAM1020, 1032, 1033, and 1034: Steroidal alkaloids (1024 mg/L), Conessine (32 mg/L); PAM1626: Steroidal alkaloids (128 mg/L), Conessine (32 mg/L)

^b^Fractional inhibitory concentration index (FIC index) where synergistic (≤ 0.5), non- synergistic (> 0.5 - ≤ 4.0), and antagonistic (> 4.0). For the strains where the steroidal alkaloids did not have a measurable MIC, the highest value tested (1024 mg/L) was used in the FICI calculation to provide a conservative estimate of the FICI value

Generally, but with some exceptions, combination of the steroidal alkaloids or conessine with the antibiotics resulted in minor reductions of the MICs of the antibiotics versus each of the *P. aeruginosa* strains tested (Table 1). However, this enhancement of antibiotic inhibition was not synergistic as none of the calculated FICI values for each of the drug combinations was ≤0.5 (Table 1).

Further study of the inhibitory action of the steroidal alkaloids or conessine in combination with levofloxacin was carried out because the fluoroquinolones are known substrates for all three of the overexpressed efflux systems studied in this work [1]. Furthermore, in a recent study that employed the same strains, levofloxacin in combination with unrelated, putative EPIs generated optimal results [21].

The effect of exposure to levofloxacin alone at MIC_0.5_, or combinations of levofloxacin with steroidal alkaloids or conessine, on the viability of the *P. aeruginosa* strains was measured using an in vitro 24 h time-kill assay (Table 2). The *P. aeruginosa* strain overexpressing the MexCD-OprJ efflux system (PAM1033) and the triple deletion strain (PAM1626) were omitted because the levofloxacin MIC of PAM1033 was unchanged by exposure to either the alkaloids or conessine, and PAM1626 is already hypersensitive to levofloxacin (Table 1).

**Table 2:** Effect of conessine and steroidal alkaloids in combination with levofloxacin on the viability of *P. aeruginosa* PAM1020, PAM1032 and PAM1034

	Strain	Agent (s)	Concentration (mg/L)	Log CFU/ml			
				Inoculum	Untreated control	Treatment (±SD)	Log reduction
Single treatment at MIC_0.5_^a^	PAM1020	Conessine	32			9.47 ± 0.03	0.27
		Alkaloids	1024	5.16 ± 0.06	9.74 **±** 0.06	8.76 ± 0.01	0.98
		LVX	0.125			8.91 ± 0.02	0.83
	PAM1032	Conessine	32			9.42 ± 0.01	0.45
		Alkaloids	1024	5.44 ± 0.08	9.87 **±** 0.14	8.82 ± 0.06	1.05
		LVX	1			8.19 ± 0.68	1.68
	PAM1034	Conessine	32			9.54 ± 0.09	0.13
		Alkaloids	1024	5.64 ± 0.21	9.67 **±** 0.02	8.88 ± 0.23	0.79
		LVX	2			8.18 ± 0.10	1.49
Combinations at MIC_0.5_	PAM1020	Conessine + LVX	32 + 0.125	5.16 ± 0.06	9.74 **±** 0.06	9.25 ± 0.03	0.49
		Alkaloids + LVX	1024 + 0.125			8.12 ± 0.16	1.62
	PAM1032	Conessine + LVX	32 + 1	5.44 ± 0.08	9.87 **±** 0.14	**5.35 ± 0.43**	**4.52**
		Alkaloids + LVX	1024 + 1			**7.70 ± 0.13**	**2.17**
	PAM1034	Conessine + LVX	32 + 2	5.64 ± 0.21	9.67 **±** 0.02	**5.54 ± 0.09**	**4.13**
		Alkaloids + LVX	1024 + 2			**6.85 ± 0.67**	**2.82**

^a^A MIC was not detectable for the steroidal alkaloids so the highest concentration tested was used in this assay

Viability was determined in 96-well microplates after 24 h exposure to the antibiotics in MHB at 37 °C. Data shown is the mean and standard deviation from quadruple experiments. Highlighted treatments are those that resulted in ≥2 log_10_ reduction compared to untreated controls

Combination of levofloxacin with either the steroidal alkaloids or conessine did enhance killing of the *P. aeruginosa* strains overexpressing MexAB-OprM and MexEF-OprN compared to the single drug treatments. The greatest enhancement of killing occurred with combinations of levofloxacin and conessine. However, similar to the findings shown in Table 1, the enhanced killing due to the combination treatments was not sufficiently potent to be termed synergistic.

In summary, combination of *H. antidysenterica* steroidal alkaloids and conessine with antibiotics resulted in enhanced inhibition in vitro of *P. aeruginosa* strains that over-express different efflux-pump systems that can confer a MDR phenotype.

### Combination treatments of levofloxacin with *H. antidysenterica* steroidal alkaloids, or conessine, show enhanced efficacy compared to the component monotherapies versus *G. mellonella* larvae infected with MDR strains of *P. aeruginosa*

The efficacy of antibiotic combinations with steroidal alkaloids or conessine were investigated in vivo using the *G. mellonella* infection model. Initially, larvae were injected with triple doses (at 2, 4 and 6 h intervals after the start of the experiment) of the steroidal alkaloids (up to 200 mg/kg) or conessine (up to 50 mg/kg) alone to determine if either were toxic and neither had any detrimental effect on the larvae after 96 h incubation at 37 °C (Additional file 1).

Preliminary studies showed that combinations of the steroidal alkaloids or conessine with the antibiotic levofloxacin gave the best results so these were investigated further in detail. Appropriate strain specific dosing regimens of levofloxacin, steroidal alkaloids and conessine were determined that provided little, or no, therapeutic benefit to larvae infected with the *P. aeruginosa* strains when administered as monotherapies (Additional file 2). Thus, any enhanced efficacy induced upon administration of combinations of the antibiotics with the steroidal alkaloids or conessine could be readily observed.

**Fig. 1:**
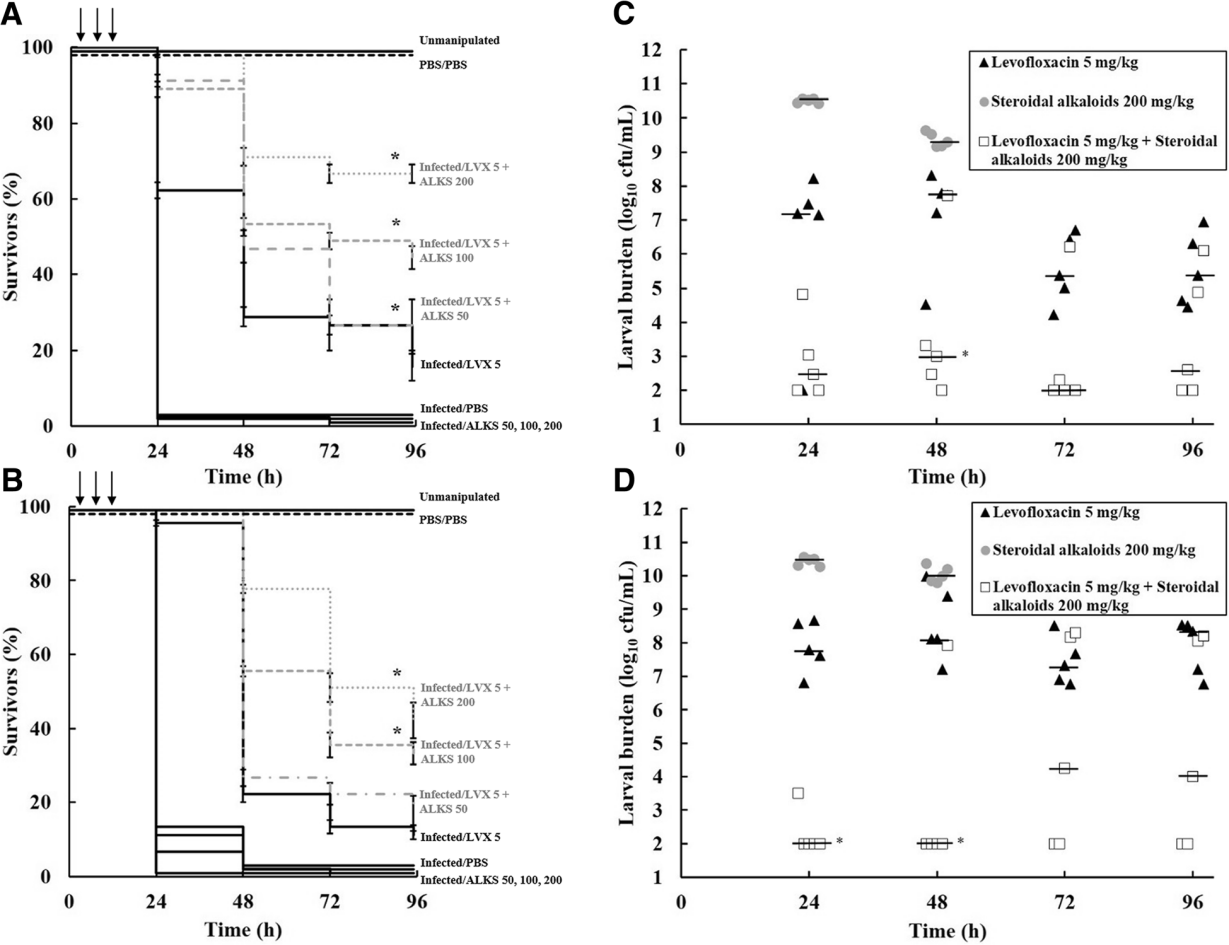
Effect of treatment with combinations of steroidal alkaloids and levofloxacin on survival of *G. mellonella* larvae infected with *P. aeruginosa* PAM1032 (**a**) and PAM1034 (**b**), or larval burden of the same strains PAM1032 (**c**) and PAM1034 (**d**). All larvae were inoculated with 2.5 × 10^3^ cfu/mL *P. aeruginosa* and treated with each agent individually or in combination with three doses at 2, 4 and 6 h post-infection (indicated by the arrows). Treatments consisted of PBS, steroidal alkaloids (50, 100 or 200 mg/kg), levofloxacin (5 mg/kg), and a combination of steroidal alkaloids with levofloxacin. Larvae were incubated at 37 °C for 96 h and survival recorded every 24 h. The burden of *P. aeruginosa* was determined from five individual larvae every 24 h. For clarity, data for treatment with PBS alone is not shown because the data obtained was similar to that obtained for steroidal alkaloid treatment alone. * **a**) and **b**); combination treatment group with significantly enhanced survival compared with any of the constituent monotherapies (*P* < 0.05, log-rank test with Holm’s correction for multiple comparisons). *n* = 45 (pooled from triplicate experiments). Error bars indicate ±SEM. LVX, levofloxacin; ALKS, steroidal alkaloids. * **c**) and **d**); significant difference in larval burden between groups treated with the combination of steroidal alkaloids and levofloxacin compared with the constituent monotherapies; *n* = 5 (*P* < 0.05, the Mann–Whitney *U*-test compared the combination therapy with each monotherapy individually). The black bar represents the median value of larval burden per group

The effect of triple doses (2, 4 and 6 h post-infection (p.i)) of levofloxacin in combination with steroidal alkaloids on the survival and bacterial burden of *G. mellonella* larvae infected with *P. aeruginosa* PAM1032 and PAM1034 is shown in Fig. 1. Treatment with triple doses of steroidal alkaloids alone (50, 100 or 200 mg/kg) had no therapeutic benefit on all of the strains tested (Fig. 1a and b). Death of the infected larvae correlated with the recovery of high numbers of *P. aeruginosa* PAM1032 or PAM1034 from within the larvae after just 24 h (Fig. 1c and d respectively). Similarly, a triple dose of levofloxacin (5 mg/kg) resulted in only a minor increase in survival of infected larvae compared to infected larvae treated with PBS (Fig. 1a and b) and the rapid growth of both *P. aeruginosa* strains within the larvae was not significantly reduced (Fig. 1c and d).

Notably, treatment with the same triple doses of the steroidal alkaloids in combination with 5 mg/kg of levofloxacin resulted in significantly enhanced survival compared to triple doses of the monotherapies (*P* < 0.05). Enhanced survival after combination therapy was most pronounced with PAM1032 infections but was also evident with PAM1034 to a lesser extent (Fig. 1a and b). Furthermore, combination therapy completely prevented the rapid proliferation of bacteria within the larvae reflected by large reductions in bacterial burden compared to that seen with the monotherapies over the duration of the experiment (Fig. 1c and d). The enhanced efficacy of levofloxacin occurred in a dose-dependent fashion increasing as the co-administered dose of the steroidal alkaloids increased. Significantly enhanced efficacy was only observed with the strains overexpressing MexAB-OprM (PAM1032) and MexEF-OprN (PAM1034). A small enhancement (*P* < 0.05) in survival was observed in the parent strain (PAM1020) but only at the highest dose of steroidal alkaloids administered (Additional file 3a). No enhancement in survival after combination therapy was observed in the strain overexpressing MexCD-OprJ (PAM1033) or the strain with the three efflux-pump systems deleted (PAM1626) (Additional file 3b and c respectively).

**Fig. 2:**
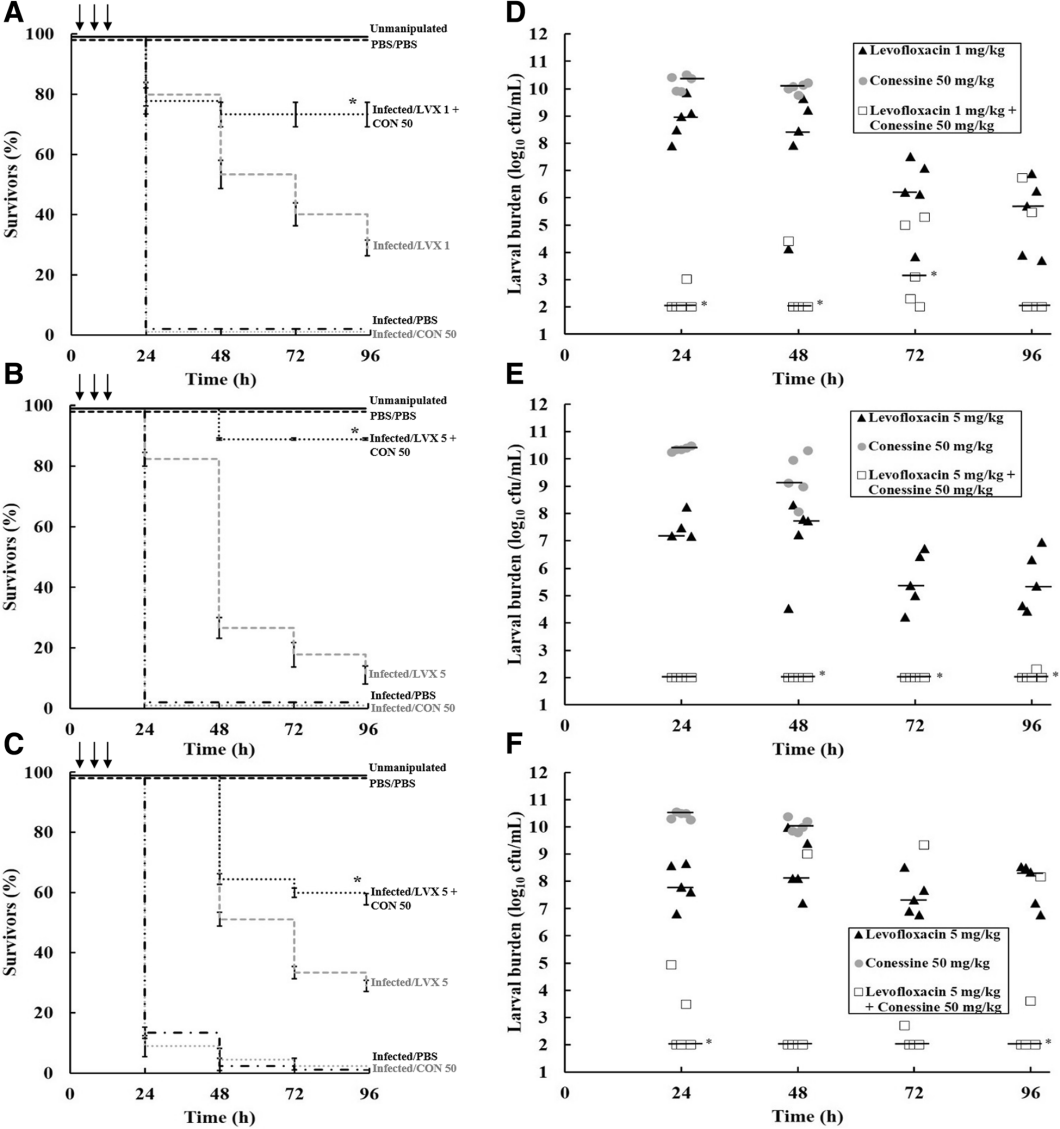
Effect of treatment with combinations of conessine and levofloxacin on survival of *G. mellonella* larvae infected with *P. aeruginosa* PAM1020 (**a**), PAM1032 (**b**) and PAM1034 (**c**), or larval burden of the same strains PAM1020 (**d**), PAM1032 (**e**) and PAM1034 (**f**). All larvae were inoculated with 2.5 × 10^3^ cfu/mL *P. aeruginosa* and treated with each agent individually or in combination with three doses at 2, 4 and 6 h post-infection (indicated by the arrows). Treatments consisted of PBS, conessine (50 mg/kg), levofloxacin (1 or 5 mg/kg, indicated on graph), and a combination of conessine with levofloxacin. Larvae were incubated at 37 °C for 96 h and survival recorded every 24 h. The burden of *P. aeruginosa* was determined from five individual larvae every 24 h. For clarity, data for treatment with PBS alone is not shown because the data obtained was similar to that obtained for conessine treatment alone. *a), **b**) and **c**); combination treatment group with significantly enhanced survival compared with any of the constituent monotherapies (*P* < 0.05, log-rank test with Holm’s correction for multiple comparisons). *n* = 45 (pooled from triplicate experiments). Error bars indicate ±SEM. LVX, levofloxacin; CON, conessine. * **d**), **e**) and **f**); significant difference in larval burden between groups treated with the combination of conessine and levofloxacin compared with the constituent monotherapies; *n* = 5 (*P* < 0.05, the Mann–Whitney *U*-test compared the combination therapy with each monotherapy individually). The black bar represents the median value of larval burden per group

Similar studies were carried out with combinations of conessine with levofloxacin rather than the steroidal alkaloids (Fig. 2). Treatment with a triple dose of conessine (50 mg/kg) alone had no therapeutic effect on larvae infected with all of the *P. aeruginosa* strains tested and did not prevent the rapid increase in bacterial burden of the inoculated *P. aeruginosa* strains that occurred after the first 24 h p.i (Fig. 2). Similarly, strain-specific, triple doses of levofloxacin alone were administered that resulted in only a small increase in survival of infected larvae compared to those treated with PBS: PAM1020–1 mg/kg (Fig. 2a) and PAM1032 or PAM1034 5 mg/kg (Fig. 2b and c, respectively). Correlating with larval survival, these doses of levofloxacin resulted in only a minor reduction in the bacterial burden within the larvae over the 96 h duration of the experiment (Fig. 2d, e and f).

Treatment with a triple dose of a combination of conessine (50 mg/kg) with the same, strain-specific levofloxacin doses mentioned previously, resulted in significantly enhanced survival compared to triple doses of the monotherapies (*P* < 0.05). Enhanced efficacy was most notable versus the *P. aeruginosa* strain overexpressing the MexAB-OprM efflux pump (PAM1032) (Fig. 2b) but the combination therapy also resulted in enhanced survival of the parent strain (PAM1020) (Fig. 2a) and the strain overexpressing MexEF-OprN (PAM1034) (Fig. 2c). As before, the combination treatment resulted in correlative reductions in bacterial burden within infected larvae over the 96 h duration of the experiment with the inhibitory effect on bacterial growth being most notable for PAM1032 (Fig. 2e) but was also evident in larvae infected with either PAM1020 (Fig. 2d) or PAM1034 (Fig. 2f). As shown with the steroidal alkaloids previously, combination therapy of conessine with levofloxacin did not result in enhanced survival of larvae infected with either PAM1033 (overexpressing MexCD-OprJ) or PAM1626 (the strain with the three efflux-pump systems deleted) (Additional file 4a and b respectively).

In summary, combination therapies of conessine or steroidal alkaloids with levofloxacin restored antibiotic efficacy in vivo versus infections with strains of *P. aeruginosa* overexpressing either the MexAB-OprM or MexEF-OprN efflux pumps. These observations were supported by the results obtained in vitro. The enhanced efficacy of the combination treatments was reflected in high levels of larval survival that correlated with reduced larval burden of the infecting pathogens. In some cases the combination treatment completely eradicated detectable bacteria within the larvae with numbers remaining below the level of detection (≤ 2 log_10_ cfu/mL).

## Discussion

Mutations that result in the over-expression of efflux pumps and confer a MDR phenotype on *P. aeruginosa* strains are frequently isolated from infected patients [1, 3]. Thus, the overexpression of efflux pumps such as MexAB-OprM, MexCD-OprJ, MexEF-OprN, and MexXY-OprM render many therapeutic options for serious *P. aeruginosa* infections redundant. As a consequence, many studies have addressed the possibility of co-administering EPIs with antibiotics to restore the clinical efficacy of antibiotics that are otherwise rendered ineffective [4, 5].

A previous study conducted in the corresponding author’s lab employed well-characterized *P. aeruginosa* strains (gifted by [22]) that over-express the RND efflux pumps in conjunction with a *G. mellonella* larva infection model [21]. Notably, this demonstrated that infection with strains that overexpress efflux pumps resulted in antibiotic treatment failure in *G. mellonella* larvae, reproducing the treatment outcomes seen in human patients, and illustrating that this invertebrate model can be employed to identify novel treatments for MDR *P. aeruginosa* infections. Subsequently, the model was used to identify putative EPI/antibiotic combinations that restored antibiotic efficacy versus efflux-pump mediated *P. aeruginosa* infections in vivo [10, 21]. The present work has used the same *P. aeruginosa* strains and presents evidence that combinations of levofloxacin with *H. antidysenterica* extract, and the principal active ingredient alone (the steroidal alkaloid conessine), show no toxicity in vivo but enhanced efficacy in vitro and in vivo and represent a novel treatment option meriting further investigation.

Available evidence suggests that *H. antidysenterica* steroidal crude extract, and conessine may be acting as EPIs [19, 20]. The steroidal extract and the alkaloid conessine have recently been reported to enhance antibiotic activity due to interference with the AdeIJK efflux pump in *A. baumannii* [19] which is functionally equivalent to the MexAB-OprM pump of *P. aeruginosa* [30]. Furthermore, conessine restored antibiotic susceptibility to an otherwise resistant *P. aeruginosa* strain that overexpressed the MexAB-OprM efflux pump [20]. The data reported in the present study does not demonstrate that either the steroidal extracts or conessine are EPIs but is consistent with this hypothesis. For example, both the steroidal extracts and conessine restored the in vivo efficacy of levofloxacin only against the parent strain (where MexAB-OprM is known to be constitutively expressed; [3]) and the strains overexpressing the MexAB-OprM or MexEF-OprN efflux pumps. They had no restorative effect on the strain overexpressing MexCD-OprJ or the strain with all three of the Mex efflux pumps deleted. This specificity for certain Mex efflux systems does imply that compounds within the extract and conessine could be acting as EPIs. Furthermore, the finding that the restorative effect on levofloxacin efficacy was much less potent with the crude extract of steroidal alkaloids compared to the principal active ingredient alone (the steroidal alkaloid conessine; [16]) also suggests that it is conessine that posesses these EPI-like properties. Nonetheless, it also cannot be discounted that the restorative effect of the extract and conessine on levofloxacin efficacy with the strains containing the *nalB* (MexAB-OprM overexpressed) or *nfxC* (MexEF-OprN overexpressed) mutations could be explained by other unknown effects of these mutations that are unconnected with the over-expression of the two Mex efflux pumps.

Irrespective of the precise mode of action, the ability of conessine to restore antibiotic efficacy versus MDR *P. aeruginosa* infections merits further investigation and development for potential clinical application. Precise evaluation of the toxicity of *H. antidysenterica* bark extracts has not been carried out in humans. A study of acute and subacute toxicity of the methanol extract of a related plant, *Holarrhena floribunda*, that also contains conessine and is widely used to treat gastrointestinal disorders in Cameroon, revealed a LD_50_ of 7 g/kg for female rats, indicating low levels of toxicity [31]. However, conessine alone was found to have an LD_50_ of 28.7 mg/kg after administration to mice intraperitoneally and was also observed to depress the heart and central nervous system [32]. Notably, the dose employed in the present study that elicited the optimal restorative effect on levofloxacin efficacy was higher at 50 mg/kg. Clearly, additional studies are required to identify if either the plant extracts or conessine alone are able to be safely used in human patients. Furthermore, showing that the steroidal extracts and conessine restore antibiotic efficacy in *G. mellonella* larvae does not mean that the same effects would be observed in mammals. Whilst this study has once again revealed the success of the *G. mellonella* infection model as a ‘first *in vivo*’ test for novel antimicrobial therapies, additional studies will also need to determine if the therapeutic benefit observed here also occurs in more traditional mammalian infection models.

## Conclusions

In summary, combination therapies of conessine or steroidal alkaloids with levofloxacin restored the efficacy of the antibiotic in vivo versus infections of *G. mellonella* larvae with strains overexpressing either the MexAB-OprM or MexEF-OprN efflux pumps. No restorative effect was observed on infections with strains overexpressing MexCD-OprJ or the strain with all three of the Mex efflux pumps deleted. The enhanced efficacy of the combination treatments was reflected in high levels of larval survival that correlated with large reductions in larval burden of the infecting pathogens.

## Additional files

Additional file 1: Raw data for steroidal alkaloid and conessine toxicity in vivo. (XLS 39 kb)

Additional file 2: Raw data for single treatments in vivo. (XLS 198 kb)

Additional file 3: Effect of treatment with combinations of steroidal alkaloids and levofloxacin on survival of *Galleria mellonella* larvae infected with *Pseudomonas aeruginosa* PAM1020 (a), PAM1033 (b) and PAM1626 (c). All larvae were inoculated with 2.5 × 10^3^ cfu/mL *P. aeruginosa* and treated with each agent individually or in combination with three doses at 2, 4 and 6 h post-infection (indicated by the arrows). Treatments consisted of PBS, steroidal alkaloids (50, 100 or 200 mg/kg), levofloxacin (0.05, 1 or 5 mg/kg), and a combination of steroidal alkaloids with levofloxacin. Larvae were incubated at 37 °C for 96 h and survival recorded every 24 h. * combination treatment group with significantly enhanced survival compared with any of the constituent monotherapies (*P* < 0.05, log-rank test with Holm’s correction for multiple comparisons). *n* = 30 (pooled from duplicate experiments). LVX, levofloxacin; ALKS, steroidal alkaloids. (PNG 117 kb)

Additional file 4: Effect of treatment with combinations of conessine and levofloxacin on survival of *Galleria mellonella* larvae infected with *Pseudomonas aeruginosa* PAM1033 (a) and PAM1626 (b). All larvae were inoculated with 2.5 × 10^3^ cfu/mL *P. aeruginosa* and treated with each agent individually or in combination with three doses at 2, 4 and 6 h post-infection (indicated by the arrows). Treatments consisted of PBS, conessine (50 mg/kg), levofloxacin (0.05 or 5 mg/kg), and a combination of conessine with levofloxacin. Larvae were incubated at 37oC for 96 h and survival recorded every 24 h. * combination treatment group with significantly enhanced survival compared with any of the constituent monotherapies (*P* < 0.05, log-rank test with Holm’s correction for multiple comparisons). *n* = 30 (pooled from duplicate experiments). LVX, levofloxacin; CON, conessine. (PNG 89 kb)

## Abbreviations

CCCP: Carbonyl cyanide m-chlorophenylhydrazone
EPI: Efflux pump inhibitor
FICI: Fractional inhibitory concentration index
LD_50_: 50% Lethal dose
MDR: Multidrug-resistant
MHB: Mueller-Hinton broth
MIC: Minimal inhibitory concentration
NMP: 1-(1-Naphthylmethyl)-piperazine
PAβN: Phenylalanyl arginyl β-naphthylamide
RND: Resistance–nodulation–division

## References

[1] Lister PD, Wolter DJ, Hanson ND (2009). Antibacterial-resistant *Pseudomonas aeruginosa*: clinical impact and complex regulation of chromosomally encoded resistance mechanisms. Clin Microbiol Rev.

[2] Piddock LJ (2006). Clinically relevant chromosomally encoded multidrug resistance efflux pumps in bacteria. Clin Microbiol Rev.

[3] Poole K (2011). *Pseudomonas Aeruginosa*: Resistance to the Max. Front Microbiol.

[4] Tegos GP, Haynes M, Strouse JJ, Khan MM, Bologa CG, Oprea TI (2011). Microbial efflux pump inhibition: tactics and strategies. Curr Pharm Des.

[5] Bohnert JA, Kern WV, Li XZ (2016). Antimicrobial drug efflux pump inhibitors. Efflux-Mediated Antimicrobial Resistance in Bacteria.

[6] Sun J, Deng Z, Yan A (2014). Bacterial multidrug efflux pumps: mechanisms, physiology and pharmacological exploitations. Biochem Biophys Res Commun.

[7] Bohnert JA, Szymaniak-Vits M, Schuster S, Kern WV (2011). Efflux inhibition by selective serotonin reuptake inhibitors in *Escherichia coli*. J Antimicrob Chemother.

[8] Piddock LJV, Garvey MI, Rahman MM, Gibbons S (2010). Natural and synthetic compounds such as trimethoprim behave as inhibitors of efflux in gram-negative bacteria. J Antimicrob Chemother.

[9] Morita Y, Nakashima K, Nishino K, Kotani K, Tomida J, Inoue M (2016). Berberine is a novel type efflux inhibitor which attenuates the MexXY-mediated aminoglycoside resistance in *Pseudomonas aeruginosa*. Front Microbiol.

[10] E B, PJ C (2016). Enhancement of antibiotic efficacy against multi-drug resistant *Pseudomonas aeruginosa* infections via combination with curcumin and 1-(1-Naphthylmethyl)-piperazine. J Antimicrob Agents.

[11] Negi N, Prakash P, Gupta ML, Mohapatra TM (2014). Possible role of curcumin as an efflux pump inhibitor in multidrug resistant clinical isolates of *Pseudomonas aeruginosa*. J Clin Diagn Res.

[12] Choudhury D, Talukdar AD, Chetia P, Bhattacharjee A, Choudhury MD (2016). Screening of natural products and derivatives for the identification of RND efflux pump inhibitors. Comb Chem High Throughput Screen.

[13] Prasch S, Bucar F (2015). Plant derived inhibitors of bacterial efflux pumps: an update. Phytochem Rev.

[14] Stavri M, Piddock LJV, Gibbons S (2007). Bacterial efflux pump inhibitors from natural sources. J Antimicrob Chemother.

[15] Chakraborty A, Brantner AH (1999). Antibacterial steroid alkaloids from the stem bark of *Holarrhena pubescens*. J Ethnopharmacol.

[16] Kumar N, Singh B, Bhandari P, Gupta AP, Kaul VK (2007). Steroidal alkaloids from *Holarrhena antidysenterica* (L.) WALL. Chem Pharm Bull.

[17] Chusri S, Siriyong T, Na-Phatthalung P, Voravuthikunchai SP (2014). Synergistic effects of ethnomedicinal plants of Apocynaceae family and antibiotics against clinical isolates of *Acinetobacter baumannii*. Asian Pac J Trop Med.

[18] Phatthalung PN, Chusri S, Voravuthikunchai SP (2012). Thai ethnomedicinal plants as resistant modifying agents for combating *Acinetobacter baumannii* infections. BMC Complement Altern Med.

[19] Siriyong T, Chusri S, Srimanote P, Tipmanee V, Voravuthikunchai SP (2016). *Holarrhena antidysenterica* extract and its steroidal alkaloid, conessine, as resistance-modifying agents against extensively drug-resistant *Acinetobacter baumannii*. Microb Drug Resist.

[20] Siriyong T, Srimanote P, Chusri S, Yingyongnarongkul B, Suaisom C, Tipmanee V (2017). Conessine as a novel inhibitor of multidrug efflux pump systems in *Pseudomonas aeruginosa*. BMC Complement Altern Med.

[21] Adamson DH, Krikstopaityte V, Coote PJ (2015). Enhanced efficacy of putative efflux pump inhibitor/antibiotic combination treatments versus MDR strains of *Pseudomonas aeruginosa* in a *Galleria mellonella in vivo* infection model. J Antimicrob Chemother.

[22] Lomovskaya O, Lee A, Hoshino K, Ishida H, Mistry A, Warren MS (1999). Use of a genetic approach to evaluate the consequences of inhibition of efflux pumps in *Pseudomonas aeruginosa*. Antimicrob Agents Chemother.

[23] Hill L, Veli N, Coote PJ (2014). Evaluation of *Galleria mellonella* larvae for measuring the efficacy and pharmacokinetics of antibiotic therapies against *Pseudomonas aeruginosa* infection. Int J Antimicrob Agents.

[24] Eliopoulous G, Moellering R, Lorian V (1996). Antimicrobial combinations. Antibiotics in Laboratory Medicine 3.

[25] White RL, Burgess DS, Manduru M, Bosso JA (1996). Comparison of three different *in vitro* methods of detecting synergy: time-kill, checkerboard, and E test. Antimicrob Agents Chemother.

[26] Krezdorn J, Adams S, Coote PJ (2014). A *Galleria mellonella* infection model reveals double and triple antibiotic combination therapies with enhanced efficacy versus a multidrug-resistant strain of *Pseudomonas aeruginosa*. J Med Microbiol.

[27] Bland JM, Altman DG (1998). Survival probabilities (the Kaplan-Meier method). Brit Med J..

[28] Bland JM (2004). The logrank test. Brit Med J.

[29] Holm S (1979). A simple sequentially rejective multiple test procedure. Scand J Stat.

[30] Damier-Piolle L, Magnet S, Bremont S, Lambert T, Courvalin P (2008). AdeIJK, a resistance-nodulation-cell division pump effluxing multiple antibiotics in *Acinetobacter baumannii*. Antimicrob Agents Chemother.

[31] Bogne KP, Penlap BV, Mbofung CM, Etoa F-X (2012). Acute and subacute toxicity of the methanol extract from *Holarrhena floribunda* G. Don (Apocynaceae). Europ. J Exp Biol.

[32] Stephenson RP (1948). The pharmacological properties of conessine, isoconessine and neoconessine. Brit J Pharmacol.

