# Supplementary figures and images for: Steroidal alkaloids and conessine from the medicinal plant Holarrhena antidysenterica restore antibiotic efficacy in a Galleria mellonella model of multidrug-resistant Pseudomonas aeruginosa infection
Source: BMC Complement Altern Med. 2018 Oct 19;18:285. doi: 10.1186/s12906-018-2348-9 (PMC6194700; doi:10.1186/s12906-018-2348-9)

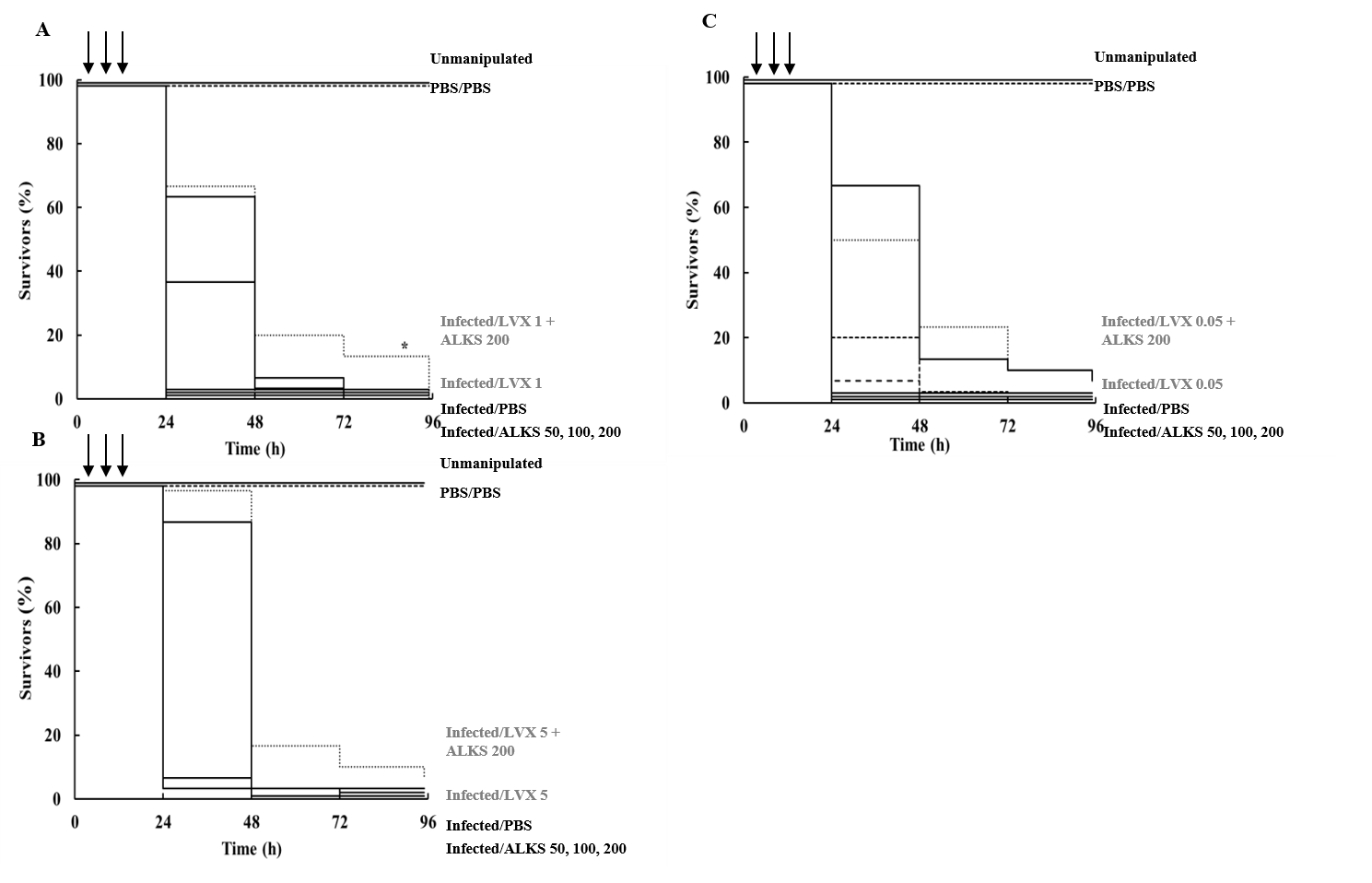

Supplement: Supplementary file 3 — Effect of treatment with combinations of steroidal alkaloids and levofloxacin on survival of Galleria mellonella larvae infected with Pseudomonas aeruginosa PAM1020 (a), PAM1033 (b) and PAM1626 (c). All larvae were inoculated with 2.5 × 10^3^ cfu/mL P. aeruginosa and treated with each agent individually or in combination with three doses at 2, 4 and 6 h post-infection (indicated by the arrows). Treatments consisted of PBS, steroidal alkaloids (50, 100 or 200 mg/kg), levofloxacin (0.05, 1 or 5 mg/kg), and a combination of steroidal alkaloids with levofloxacin. Larvae were incubated at 37 °C for 96 h and survival recorded every 24 h. * combination treatment group with significantly enhanced survival compared with any of the constituent monotherapies (P < 0.05, log-rank test with Holm’s correction for multiple comparisons). n = 30 (pooled from duplicate experiments). LVX, levofloxacin; ALKS, steroidal alkaloids. (PNG 117 kb) [file 12906_2018_2348_MOESM3_ESM.png]

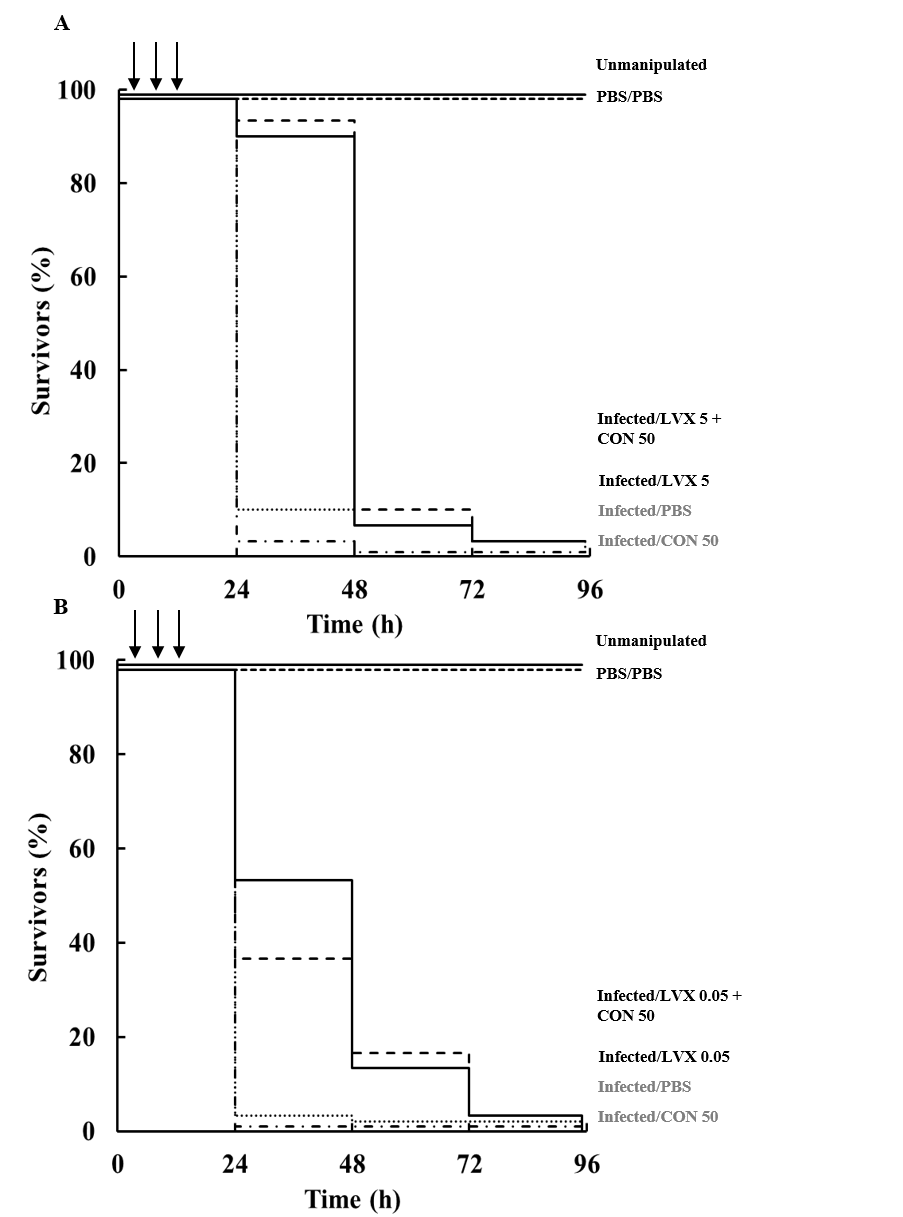

Supplement: Supplementary file 4 — Effect of treatment with combinations of conessine and levofloxacin on survival of Galleria mellonella larvae infected with Pseudomonas aeruginosa PAM1033 (a) and PAM1626 (b). All larvae were inoculated with 2.5 × 10^3^ cfu/mL P. aeruginosa and treated with each agent individually or in combination with three doses at 2, 4 and 6 h post-infection (indicated by the arrows). Treatments consisted of PBS, conessine (50 mg/kg), levofloxacin (0.05 or 5 mg/kg), and a combination of conessine with levofloxacin. Larvae were incubated at 37oC for 96 h and survival recorded every 24 h. * combination treatment group with significantly enhanced survival compared with any of the constituent monotherapies (P < 0.05, log-rank test with Holm’s correction for multiple comparisons). n = 30 (pooled from duplicate experiments). LVX, levofloxacin; CON, conessine. (PNG 89 kb) [file 12906_2018_2348_MOESM4_ESM.png]
